# A modified risk score in one-year survival rate assessment of group 1 pulmonary arterial hypertension

**DOI:** 10.1186/s12890-018-0712-7

**Published:** 2018-10-16

**Authors:** Wei Xiong, Yunfeng Zhao, Mei Xu, Bigyan Pudasaini, Xuejun Guo, Jinming Liu

**Affiliations:** 1 Department of Respiratory Medicine, Xinhua Hospital, Shanghai Jiaotong University School of Medicine, 1665, Kongjiang Road, Shanghai, 200092 China; 2 Department of Cardiopulmonary Circulation, Shanghai Pulmonary Hospital, Tongji University School of Medicine, Shanghai, People’s Republic of China; 3 Department of Respiratory Medicine, Punan Hospital, Pudong New District, Shanghai, China; 4 Department of Pediatrics, Kongjiang Hospital, Yangpu District, Shanghai, China; 5 Department of Pediatrics, Shanghai Dinghai Community Health Service Center, Tongji University School of Medicine, Yangpu District, Shanghai, China

**Keywords:** Pulmonary arterial hypertension, Group 1, Risk assessment score, Survival rate, Modified, REVEAL

## Abstract

**Background:**

Risk assessment of pulmonary arterial hypertension (PAH) contributes to its management. Unfortunately, the existing risk assessment approaches are defective for clinicians to practice in daily clinical settings to some extent.

**Methods:**

We designed a modified Risk Assessment Score of PAH (mRASP) comprising four non-invasive variables which were World Health Organization functional class(WHO FC), 6-min walk distance (6MWD), N-terminal of the pro-hormone brain natriuretic peptide(NT-pro BNP), and right atrial area(RAA), then validated it in the prediction of one-year survival rate for patients with PAH by contrast with the REVEAL risk score.

**Results:**

For the validation cohort(*n* = 216), the predicted one-year survival rate were 95–100%, 90–95%, and < 90% in the mRASP risk score strata of 0–2, 3–5, and 6–8, respectively; meanwhile, the observed one-year survival rates were 97.1, 92.6, and 52.2%, in each corresponding stratum, respectively. The mRASP (c-index = 0.727) demonstrated similar predictive power in contrast with the REVEAL risk assessment score (c-index = 0.715) in the prediction of one-year survival rate.

**Conclusion:**

The mRASP is an eligible risk assessment tool for the prognostic assessment of PAH. In contrast with the REVEAL score, it demonstrated similar predictive power and accuracy, with extra simplicity and convenience.

## Background

Pulmonary arterial hypertension is a pathophysiological disorder complicating both of cardiovascular and respiratory diseases. It is defined by a mPAP ≥ 25 mmHg, a pulmonary artery wedge pressure (PAWP) ≤ 15 mmHg and a pulmonary vascular resistance(PVR) > 3 Wood units (WU) without other causes of pre-capillary PH [1, 2].

The 2015 ESC/ERS(European Society of Cardiology/European Respiratory Society) PH guidelines strongly recommend a comprehensive regular assessment of patients with PAH since there is no single variable that provides sufficient diagnostic and prognostic information instead of a multidimensional approach [1, 3]. Based on the evaluation of multiple variables, PAH patients can be categorized as low, intermediate or high risk with estimated one-year mortality of < 5%, 5–10% and > 10%, respectively [3]. The basic program should include the functional class(FC) and at least one measurement of exercise capacity. It is also recommended to obtain some information on right ventricular (RV) function [1].

However, an individual patient is unlikely to have all variables indicative of merely one strata, thus the physician’s decision on the overall risk is subjective and the assessment could vary between different physicians [3]. One approach which can distinctly classify the risk strata of PAH is the French registry risk equation, which unfortunately concerns sex, 6MWD and cardiac output merely [3, 4]. Another one is the risk assessment score of Registry to Evaluate Early and Long-Term Pulmonary Arterial Hypertension Disease Management (REVEAL) which is a simplified risk score based on the prognostic equation, and is designed to be simple and easy enough to be adopted in everyday clinical practice, compared with the relatively complex REVEAL risk equation [5]. However, RHC is not readily available or accessible or suitable or acceptable for every patient anytime. Besides, regardless of the right atrial pressure(RAP), cardiac index (CI) and mixed venous oxygen saturation (SvO2) assessed by right heart catheterization (RHC) being the most robust indicators of RV function and prognosis, and providing important prognostic information both at the time of diagnosis and during follow-up, whereas mPAP in RHC provides little prognostic information [6–10]. In addition, some of the variables in the REVEAL risk score, such as age and PAH etiology, are not modifiable offsetting the change of modifiable variables, and potentially leading to an inaccurate evaluation of the patient’s prognosis [3]. The last but not least, the variable of vital signs such as resting systolic BP and heart rate in the REVEAL risk score is inconsistent and unreliable. Consequently, we postulated whether a modified risk assessment score could be the better approach for the prognostic assessment of PAH.

## Methods

### Study design

This study was launched in May, 2016. The eligible patients registered between May, 2014 and May, 2015 of Department of Cardiopulmonary Circulation, Shanghai Pulmonary Hospital were enrolled into the establishment cohort which was used to establish the model of mRASP by means of retrospectively corresponding the patients’ score of the mRASP in May, 2015 to the actually observed survival rate between May, 2015 and May, 2016. After the establishment of the model of mRASP, all eligible patients registered between May, 2015 and May, 2016 were enrolled into the validation cohort which was assessed by the mRASP score in May, 2016 and the REVEAL score in May, 2016, respectively and simultaneously. Patients in the validation cohort were predicted to be in certain risk strata by both risk assessment scores, then were prospectively followed up and observed for all-cause mortality in the coming 12 months till May, 2017. During the follow-up, as per their condition, all patients received at least one of the specific drug therapies available in Chinese market including: endothelin receptor antagonists: ambrisentan, bosentan; phosphodiesterase type 5 inhibitors: sildenafil, tadalafil; prostacyclin analogues: treprostinil; and calcium channel blockers(for responders of acute vasodilator testing), on the basis of supportive therapies such as oral anticoagulants, diuretics, oxygen therapy, digoxin, etc., according to the guidelines [1]. After the follow-up, for patients in each stratum stratified with the risk assessment score, the predicted one-year survival rates were validated by contrast with the actually observed one-year survival rates to explore the goodness of fit between them. Meanwhile, the predictive efficiency was compared between the mRASP score and the REVEAL score. All variables in the assessment were obtained within 3 months prior to the time point of assessment. The death of the patients who died out of hospital was confirmed by telephone follow-up at the end of every month. This protocol was approved by the Institutional Review Board of Shanghai Pulmonary Hospital. Written informed consents were obtained from all eligible patients enrolled in this study.

### Study population

All eligible patients were enrolled according to the inclusion and exclusion criteria. Inclusion criteria:1) age ≥ 18 years; 2) a diagnosis of PH on the presence of mPAP ≥ 25 mmHg and PAWP ≤15 mmHg and a PVR > 3 Wood units (WU) in RHC. Exclusion criteria: 1)a diagnosis of PH in Group 2, Group 3, Group 4, and Group 5 according to classifications in 2015 ESC/ERS PH guidelines [1]; 2) co-morbidity with other severe cardiopulmonary diseases; 3)absence of any variables involved in risk assessment; 4) no adherence to PAH-specific therapy; 4) loss to follow-up.

### Risk assessment tools

#### The REVEAL risk assessment score

Variables independently associated with increased mortality by physical examination or laboratory tests: men aged > 60 years, PAH associated with portal hypertension, PAH associated with connective tissue disease, family history of PAH, modified New York Heart Association (NYHA)/World Health Organization (WHO) functional class III or IV, renal insufficiency, resting systolic BP < 110 mmHg, heart rate > 92 beats/min, mean RAP > 20 mmHg, 6MWD < 165 m, NT-pro BNP > 1500 pg/mL, PVR > 32 WU, % predicted diffusing capacity of lung for carbon monoxide (Dlco) ≤32%, and the presence of pericardial effusion on echocardiogrphy. Risk strata are indicated by the lines: predicted one-year survival is 95 to 100% in the low-risk group, 90 to 95% in the average-risk group, 85 to 90% in the moderately high-risk group, 70 to 85% in the high-risk group, and < 70% in the very high-risk group.The average predicted one-year survival is 95 to 100% (low-risk) for patients with risk scores of 0 to 7. Similarly, the ranges specified for average-risk, moderately high-risk, high-risk, and very high-risk correspond to risk scores of 8, 9, 10 to 11, and ≥ 12, respectively [5].

#### The modified risk assessment score

**Fig. 1:**
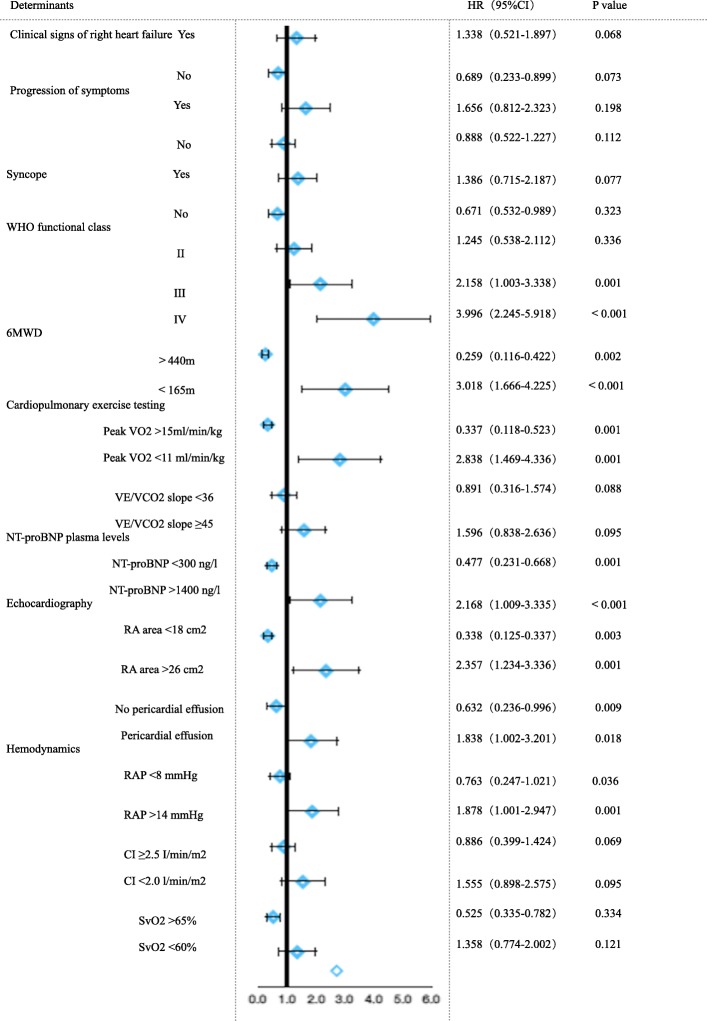
The multivariate analysis between all the risk assessment determinants in 2015 ESC/ERS PH guidelines and the risk of one-year mortality in the establishment cohort

We conducted an univariate and then a multivariate analysis between all the determinants in the TABLE 13 of 2015 ESC/ERS PH guidelines and the risk of one-year mortality. The results showed that one-year mortality was mostly correlated with 4 noninvasive variables which were WHO FC, 6MWD, NT-pro BNP, and right atrial area(RAA) in echocardiography. Figure 1 The modified risk assessment score of PAH (mRASP) consists of four variables which are WHO FC, 6MWD, NT-pro BNP, and right atrial area(RAA) in echocardiography. For each variable, the specification was derived from the variable in the TABLE 13 of 2015 ESC/ERS PH guidelines [1]. The specific feature of mRASP is that each column of low-risk, intermediate-risk, and high-risk in it account for a score of 0 point, 1 point, and 2 points, respectively. The total score of mRASP ranged between a minimum of 0 and a maximum of 8. The algorithm of the mRASP was generated by retrospectively corresponding the patients’ score of the mRASP to the actually observed survival rate in the establishment cohort. The results showed that the risk score stratum of 0–2, 3–5, and 6–8 in mRASP corresponded to the low-risk class in which survival rate was 95 to 100%, intermediate-risk class in which survival rate was 90 to 95%, and high-risk class in which survival rate was < 90%, respectively. The mRASP form is illustrated in Table 1.

**Table 1:** The modified risk assessment score of PAH

Determinants of prognosis	Score 0	Score 1	Score 2
WHO functional class	I, II	III	IV
6MWD	> 440 m	165-440 m	< 165 m
NT-proBNP	NT-proBNP < 300 ng/l	NT-proBNP 300–1400 ng/L	NT-proBNP > 1400 ng/L
Echocardiography	RA area < 18 cm^2^	RA area 18–26 cm^2^	RA area > 26 cm^2^
Total score	0–2 → Low-risk stratum → One-year survival rate 95–100%		
	3–5 → Intermediate-risk stratum→One-year survival rate 90–95%		
	6–8 → High-risk stratum→One-year survival rate < 90%		

*PAH* pulmonary arterial hypertension, *WHO* world health organization, *6MWD* 6-min walking distance, *BNP* brain natriuretic peptide, *RA* right atrial

Overall, REVEAL score comprises 9 variables including non-modifiable one such as etiological subgroup, invasive one such as RHC, and some inconsistent one such as vital signs, whereas mRASP score comprises 4 noninvasive variables in REVEAL score: WHO FC, 6MWD, NT-pro BNP and echo. WHO FC was assessed by the patients’ attending physicians who had abundant clinical experience in the management of PAH. 6MWD and echocardiography were performed by professional personnel under standard operating procedure.NT-pro BNP was assayed with AQT90 FLEX rapid immune analyzer of Radiometer Medical ApS. Echocardiography was conducted with GE VIVID i color Doppler ultrasonography.

### Statistical analysis

On the basis of the prevalence of group 1 PAH in China, to obtain a two-sided 95% confidence interval of 2.5% for the prevalence of group 1 PAH, we estimated that a sample size of 200 patients with group 1 PAH should be required. Calibration plot was used as an approach to show agreement between the mean predicted one-year survival rates beforehand and the actually observed survival rates afterwards in the validation cohort to validate the validity of risk assessment score. The mean predicted one-year survival rate was defined as the mean value of each risk stratum. That is to say, for mRASP score, the mean predicted survival rates were 97.5, 92.5 and 45.0% in low-risk, intermediate-risk and high-risk stratum, respectively. Likewise, for the REVEAL score, the mean predicted survival rates were 97.5, 92.5, 87.5, 77.5, and 35.0% in low-risk, average-risk, moderately high-risk, high-risk, and very high-risk stratum, respectively. Comparison of predictive power between the mRASP score and the REVEAL score was assessed by c-index which means the probability of concordance signifying an approach of how significant the predictive model distinguishes patients who survive from who die, and of how much the chance that the patient with lower predicted risk score will survive longer. A *p*-value < 0.05 was considered to have statistical significance.

## Results

### Demographics and characteristics of patients in two cohorts

By the time of May, 2015, 108 patients with PAH from May, 2014 to May, 2015 was determined to be the establishment cohort. Between May, 2015 and May, 2016, 18 patients died and 5 patients lost to follow-up in this cohort. For the validation cohort, it comprised 216 patients besides 7 cases who were lost to the follow-up between May, 2016 and May, 2017. The mean age, proportion of female patients, 6MWD, NT-pro BNP, and proportion of WHO FC III or IV of establishment cohort and validation cohort were 52.8 and 54.6(*p* = 0.088), 71.3 and 73.6%(*p* = 0.123), 338 m and 309 m(*p* = 0.019), 3268 pg/mL and 3497 pg/mL(*p* = 0.066), 63.9% and 72.2%(*p* = 0.005), respectively. The demographics and characteristics of patients in the establishment cohort and the validation cohort are summarized in Table 2.

**Table 2:** Characteristics of patients in two cohorts

Characteristics	Establishment cohort (*n* = 108)	Validation cohort (*n* = 216)	*p* Value
Age-years	52.8 ± 14.9	54.6 ± 17.2	0.088
Female-no.(%)	77 (71.3)	159 (73.6)	< 0.001(0.123)
WHO group 1 subgroup-no.(%)			
Idiopathic PAH	48 (44.4)	100 (46.3)	< 0.001(0.147)
Associated with CTD	35 (32.4)	62 (28.7)	< 0.001(0.007)
Associated with CHD	10 (9.3)	25 (11.6)	< 0.001(0.358)
Associated with PoPH	7 (6.5)	10 (4.6)	0.086(0.414)
Familial PAH	3 (2.8)	6 (2.8)	< 0.001(0.95)
Other	5 (4.6)	13 (6.0)	< 0.001(0.27)
WHO functional class-no.(%)			
I	9 (8.3)	21 (9.7)	< 0.001(0.33)
II	30 (27.8)	39 (18.1)	0.046(0.007)
III	55 (50.9)	131 (60.6)	< 0.001(0.025)
IV	14 (13.0)	25 (11.6)	< 0.001(0.259)
Systolic BP-mm Hg	122 ± 23	115 ± 19	0.005
Heart rate-beats/min	85 ± 15	88 ± 17	0.151
6MWD-m	338 ± 119	309 ± 125	0.019
N-terminal proBNP -pg/mL	3268 ± 2431	3497 ± 2896	0.066
Renal insufficiency(yes)-no.(%)	11 (10.2)	19 (8.8)	0.006(0.382)
DLco of predicted in PFT -%	53.8 ± 24.9	49.7 ± 22.3	0.024
Peak VO2 in CPET -ml/min/kg	14.4 ± 9.2	12.8 ± 8.6	0.036
Echocardiography			
Right atrial area-cm^2^	21.6 ± 14.9	23.1 ± 16.7	0.188
Pericardial effusion(yes)-no.(%)	28 (25.9)	66 (30.6)	< 0.001(0.033)
Right heart catheterization			
Right atrial pressure-mm Hg	9.7 ± 5.2	10.6 ± 5.8	0.234
Mean pulmonary artery pressure-mm Hg	45.2 ± 12.8	46.7 ± 13.4	0.371
Pulmonary vascular resistance-WU	10.5 ± 5.1	11.2 ± 6.3	0.565
Death -no.(%)	18(16.7)	40(18.5)	< 0.001(0.096)
Loss to follow-up -no.(%)	5(4.6)	7(3.2)	0.071(0.122)

*WHO* world health organization, *PAH* pulmonary arterial hypertension, *CTD* connective tissue diseasem, *CHD* congenital heart disease, *PoPH* portal pulmonary hypertension, *6MWD* 6-min walking distance, *BNP* brain natriuretic peptide, *DLco* diffusing capacity of lung for carbon monoxide, *PFT* pulmonary function test, *VO2* volume of oxygen, *CPET* cardiopulmonary exercise testing, *WU* Wood units

### Model establishment of the mRASP

**Fig. 2:**
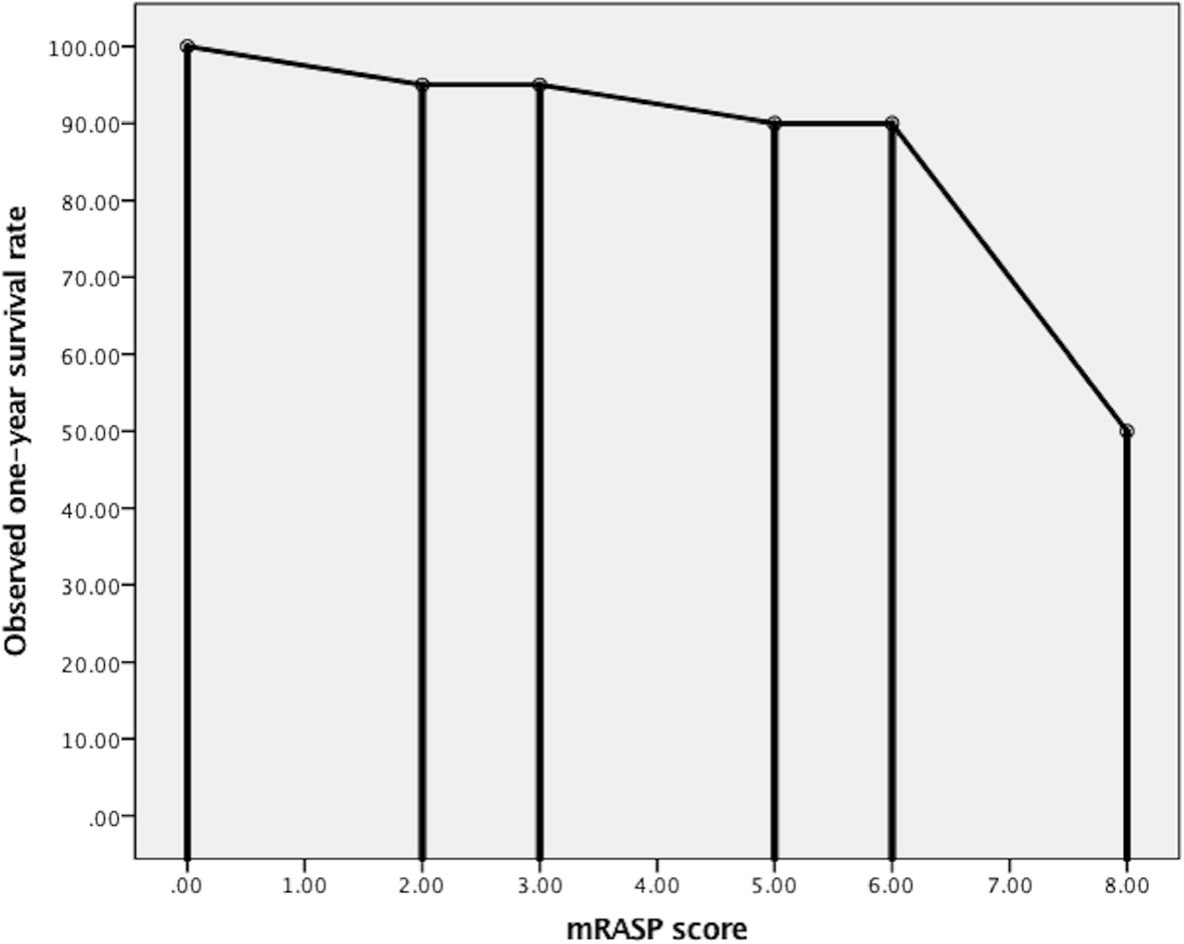
The retrospective correspondence between the mRASP score and the observed survival rates in the establishment cohort

The model of modified risk assessment score of PAH was established by using the establishment cohort. The score of mRASP of establishment cohort had a minimum risk score of 0, a maximum risk score of 8, and a mean risk score of 4.5. After the retrospective correspondence between the risk score and the observed survival rates in the establishment cohort, a score of 0–2, 3–5, and 6–8 were corresponded to the low-risk stratum in which survival rate was 95 to 100%, the intermediate-risk stratum in which survival rate was 90 to 95%, and the high-risk stratum in which survival rate was < 90%, respectively Fig. 2.

### Validation of the mRASP

In the validation cohort, the observed mean overall one-year survival rate was 81.5%. In total of 40 patients died in the follow-up. Among all the deceased, 29 cases died of aggravation of right ventricular failure during hospitalization, 11 cases died of sudden death out of hospitalization.

**Fig. 3:**
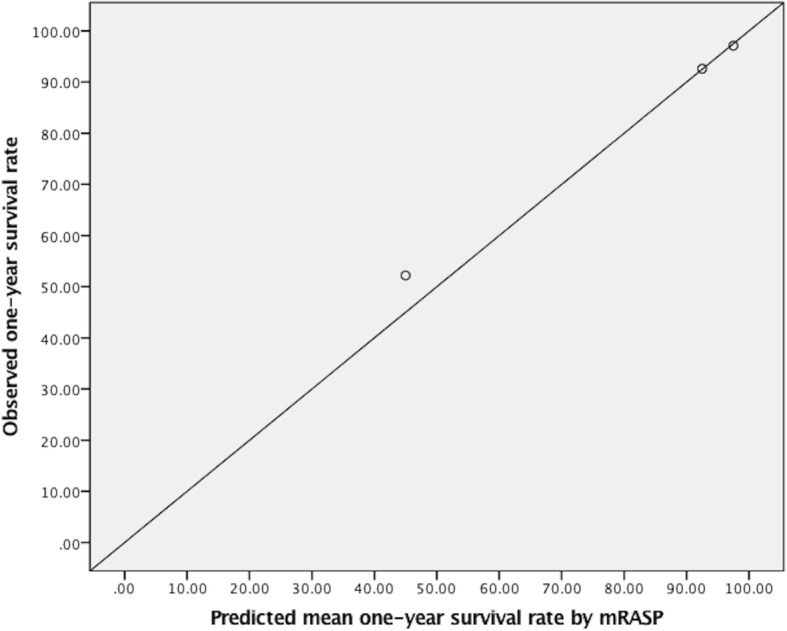
Calibration plot between the predicted mean one-year survival rates and the observed survival rates by the mRASP score in the validation cohort

On the basis of the patients’ mRASP scores being 0–2, 3–5, and 6–8, they were stratified into low-risk stratum, intermediate-risk stratum and high-risk stratum in which the predicted one-year survival rates were expected to be 95–100%, 90–95%, and 0–90%, respectively. The number of patients in low-risk stratum, intermediate-risk stratum, and high-risk stratum in the validation cohort were 68, 81, and 67 presenting in normal distribution. During the follow-up, the number of the deceased patients in each risk stratum were 2, 6, and 32, respectively. The observed one-year survival rates in each risk stratum were 97.1, 92.6, and 52.2%, respectively. The observed survival rates fell within the range of the pre-estimated risk strata. Calibration plot between the predicted mean one-year survival rates by the mRASP score and the actually observed survival rates in the validation cohort is illustrated in Fig. 3.

**Fig. 4:**
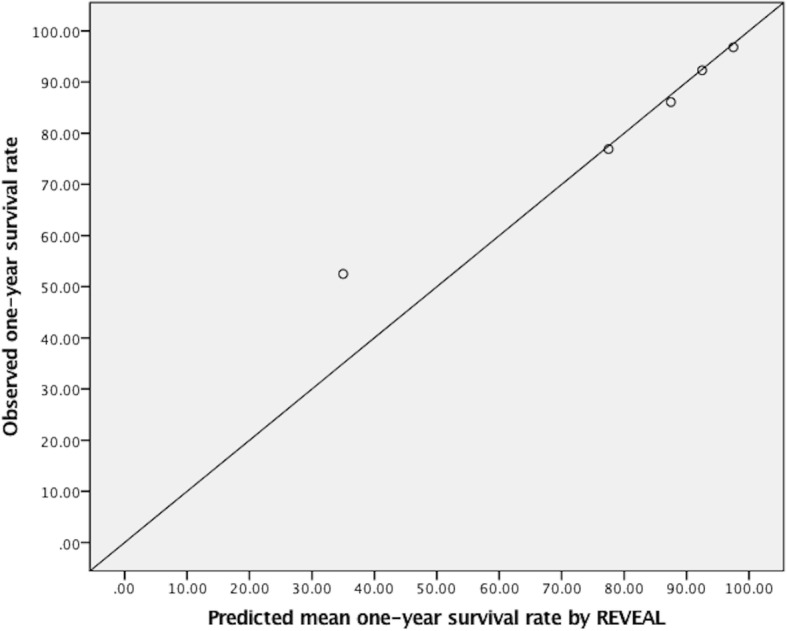
Calibration plot between the predicted mean one-year survival rates by the REVEAL score and the observed survival rates in the validation cohort

For the REVEAL score, the number of patients stratified into the REVEAL score strata of 0–7, 8, 9, 10–11, and ≥ 12 in the validation cohort were 62, 26, 36, 52, and 40, respectively. During the follow-up, the number of the deceased patients in each risk stratum were 2, 2, 5, 12 and 19, respectively. The observed one-year survival rates were 96.8, 92.3, 86.1, 76.9 and 52.5% in each risk stratum, respectively. The observed survival rates all fell within the range of the pre-estimated risk stratum 95–100%, 90–95%, 85–90%, 70–85%, and < 70%. Calibration plot between the predicted mean one-year survival rates by the REVEAL score and the actually observed survival rates in the validation cohort is illustrated in Fig. 4.

### Comparison of predictive power between the REVEAL score and the mRASP score

The bias-corrected c-index for the mRASP score in the validation cohort was calculated to be 0.727. The bias-corrected c-index for the REVEAL score in the validation cohort was 0.715. That is to say, patients with lower predicted risk score of the mRASP and the REVEAL will have 72.7 and 71.5% chances to survive longer, respectively(*p* = 0.666). The similar c-index of both risk scores indicated that the mRASP score had similar discriminatory ability with the REVEAL score.

## Discussion

Clinical daily assessment is a critical method to evaluate patients with PH, for determining disease severity and prognosis as well as disease management, and should be performed regularly with a combination of variables [1, 3]. Although the existing risk assessment approaches have been validated to be valid in predicting survival rates in multiple cohorts [4, 5, 11], there are still some defectives which reserved potential rooms for improvement. In consequence, this study was aimed at developing a modified risk assessment score of PAH. For the model establishment of this modified risk assessment score of PAH, we deliberated our desirable determinants on the principle of validity, accuracy, simplicity and convenience. The incentives that we applied those four determinants to be the variables of our modified risk assessment score was not only that they were the basic program most frequently used in PH centers [1], but also they were qualified to be mostly correlated with mortality in a multivariate analysis. Also the score provided quantitative assessments rather than qualitative ones, since the latter might vary dramatically between physicians [12].

WHO FC is one of the most valid predictors of survival, for both diagnosis and follow-up notwithstanding its variability [6, 7, 13, 14]. A deteriorating FC is one of the most serious sign of disease progression [7, 8]. 6MWD is the result of 6-min walking test (6MWT) which is a sub-maximal exercise test. It is the most inexpensive and familiar exercise test frequently used in PH centers. The overall treatment goal for patients with PAH is to achieve a low-risk status which usually means being in WHO-FC II, mostly together with a normal or near normal 6MWD [1, 15–20]. BNP/NT-proBNP levels represent myocardial dysfunction and provide prognostic information at the time of diagnosis or during follow-up [21]. NT-proBNP is regarded as a stronger predictor of prognosis compared with BNP [22]. Echocardiography is an important follow-up approach due to RV function is a crucial determinant of outcome in PH [1]. On the contrary, clinical signs of right heart failure, progression of symptoms, syncope and pericardial effusion of echocardiography in which all the severity are difficult to stratify were excluded from the mRASP in order to improve accuracy. Since life expectancy has been improved for patients with PAH warranting noninvasive approaches for prognostic assessment, and there has been no evidence that receiving regular RHC is associated with better outcomes than a non-invasive follow-up strategy [1], HC was not included in the mRASP. The reason we did not use the serial risk score assessments was that it involved the variables of two different time points resulting in the poor feasibility of the assessment of newly diagnosed patients or patients whose last assessment scores are not available [23].

The results of the present study demonstrated that the one-year survival rates predicted by the mRASP matched the actually observed ones. This validated the validity of mRASP which was derived from the establishment cohort for assessing one-year survival rates in the validation cohort. In our opinion, a valid risk assessment score of PAH should have excellent applicability, generalizability and adaptability for PAH cohorts with various characteristics, and outstanding discriminatory power to distinguish the potential survival from mortality. We noticed that patients in the validation cohort appeared to be more severe than those in the establishment cohort in regard to WHO FC, and believed that it was due to the validation cohort had more newly diagnosed patients who had never received any PAH-specific therapy than the establishment cohort. Nevertheless, from another perspective, it reveals the excellent applicability, generalizability and adaptability of the mRASP in different cohorts with different severity. It is worth noting that otherwise than the study of Benza et al. [5] there is a plunge of survival rate between the intermediate-risk stratum and the high-risk stratum in the validation cohort similar to what happened in the establishment cohort. Between score of 5 points and 6 points, the survival rate descends from more than 90% to almost its half, meanwhile, the mortality rate ascends approximately 6 folds. It may suggest that a score of 6 points could potentially be a cutoff value which implies the prognosis may deteriorate dramatically if patients’ risk scores exceed it, distinctly differentiating the potential survival from mortality.

The next comparison showed that the predictive efficacy for one-year survival rate by the mRASP score was similar to that by the REVEAL score. In the study of Benza et al. in 2012 [5], the REVEAL simplified risk score calculator were demonstrated to have good discriminatory power in patients with PAH. Afterwards, this risk assessment tool has been validated to be effective in the prediction of survival in several cohorts, demonstrating its prognostic generalizability in different PAH populations [4, 11]. It is the mostly recognized risk assessment score for PAH to date. Nevertheless, due to some problematic issues we encountered in the application of REVEAL score such as the poor accessibility of RHC, instability of vital signs, non-modifiable determinants, inspiring us to search for some solution through this study. The conception of the mRASP was an overlapping of determinants in the TABLE 13 of 2015 ESC/ERS PH guidelines and the score calculator of the REVEAL score. The original purpose of designing was aimed to inherit their pros and discard their cons to generate a simplified standardized algorithm which could be highly applicable and valid under most circumstances by means of validating the generalizability of those cut-off values in parameters from expert opinion or consensus which might be highly representative. Also the four selected variables was validated to be mostly correlated with mortality in a multivariate analysis. Since finally the two risk assessment tools did not display much distinction on validity from each other, the mRASP could be regarded as a risk assessment model with noninvasiveness, accuracy, simplicity, and convenience comparable with the REVEAL score.

Regardless of the advantages that the mRASP has, several issues must be addressed for its clinical application. It is noteworthy that since RHC is absent in the mRASP, clinicians should apply it with discretion whilst therapeutic decisions can be generated from the results [1]. It also should be noted that even though the mRASP may provide prognostic information to guide therapeutic decisions, the individual application must be performed carefully in light of that it is too population-based to precisely predict individual patient, being similar to the REVEAL score or French risk equation. In other words, when it comes to an individual patient, all risk assessments should be applied under the circumstances of considering the patient’s history and the corresponding PAH-specific therapy. Another important issue is that patients should not calculate their risk themselves for avoiding the misinterpretation. It is the responsibility of medical professionals to discuss the results of risk assessment and to consider the next steps [3]. Another issue that cannot be overlooked is that even though we endeavored to optimize the designing of mRASP as much as possible, it is potentially possible that other designs can achieve the same or even better assessment effect. For example, recently Boucly et al. built a risk assessment model composed of the following determinants: WHO FC I or II, 6MWD > 440 m, RAP < 8 mmHg and CI ≥ 2.5 L·min^− 1^·m^− 2^, which could accurately predict the prognosis of incident PAH in a retrospective study [24]. However, as said in the article, it remained unknown whether the addition of echocardiography or cardiopulmonary exercise testing to their criteria could further improve the prognostic power [24]. In another study, Hoeper et al. validated the validity of risk assessment strategy in 2015 European PH guidelines with a model composed of WHO FC, 6MWD, BNP or NT-pro BNP, right atrial pressure, cardiac index and mixed venous oxygen saturation [25]. However, this model is still invasive not being suitable for daily clinical practice. In any case, novel assessment models invariably require prospective validation. As our recognition and management of PAH advances, predictive tools will need updating to reflect current practice [4].

The strength of this study is that we prospectively validated the validity of the newly designed risk score, by contrast with the REVEAL score. Nevertheless, limitations also exist in this study. First of all, the sample volume is not very large. The validation of the mRASP in another large cohort is warranted in the future. Secondly, in the development of mRASP score by retrospectively reviewing the establishment cohort, the risk score-related assessments were not performed at mandatory visits undermining the quality of the study more or less. Thirdly, since this study did not involve the predictive efficacy of the mRASP for the survival rate beyond 1 year or for the survival rate in other groups of PH, we have no comments to make on it. The study regarding the long-term risk assessment of PAH or of other groups of PH with the mRASP is warranted in the future.The last but not least, since all patients assessed in our cohorts were all of Chinese population, this risk assessment score may not be applicable for other races.

## Conclusions

In conclusion, under the circumstances of existing risk assessment approaches for PAH having limitations in daily clinical practice, a modified risk assessment score of PAH was designed in order to improve it. The mRASP was validated to be an eligible risk assessment tool for the prognostic assessment of PAH. It demonstrated the similar predictive power to the REVEAL score in the validation of predicting one-year survival rates for patients with newly and previously diagnosed PAH. Along with its noninvasiveness, accuracy, simplicity and convenience, the mRASP may be a substitute for the REVEAL score under some circumstances. Although the mRASP still needs to further prove its consistency and stability in the future, we hope that it would at least contribute an inspiration to clinicians in the risk assessment of PAH.

## Abbreviations

6MWD: 6 min walk distance
CAT: COPD assessment test
CPET: Cardiopulmonary exercise test
CTEPH: Chronic thromboembolic pulmonary hypertension
mPAP: Mean pulmonary arterial pressure
mPAP: Mean pulmonary arterial pressure
NT-proBNP: N-terminal pro-brain natriuretic peptide
PAH: Pulmonary arterial hypertension
PAH: Pulmonary arterial hypertension
PAWP: Pulmonary artery wedge pressure
Peak VO_2_: peak oxygen consumption
PFT: Pulmonary function test,
RBT: Routine blood test
RHC: Right heart catheterization
RV: Right ventricular
